# The synthesis of water-soluble CDM-AM copolymer by irradiation and its solubilization effect on hydrophobic drugs

**DOI:** 10.1080/15685551.2018.1480681

**Published:** 2018-06-25

**Authors:** Yong-Fu Li, Hong-Tao Tang, Si-Jing Zhou

**Affiliations:** a Institute of Integragted Agricultural Development, Guizhou Academy of Agricultural Sciences, Guiyang, Guizhou, China; b National Center for Child Nutriment Quality Supervision and Testing, China National Children’s Center, Beijing, China; c Institute of Radiation Microbiology, Beijing Radiation Center, Beijing, China

**Keywords:** Irradiation, Solubility, β-cyclodextrin, Copolymer, Fungicidal activity

## Abstract

The β-cyclodextrin-acrylamide (CDM-AM) copolymer was prepared from acrylamide and β-CD maleate (CDM) using ^60^Co γ-ray irradiation method. The optimized preparation conditions for the CDM-AM copolymer are as follows: CDM:AM mass ratio of 1:1; irradiation dose of 4 kGy; and using 20 mL of DMF water solution. The yield rate of CDM-AM was 75% in grams using these synthetic conditions. The effects of the CDM-AM copolymer on the solubility and fungicidal activity of natamycin (NM) and carbendazim (MBC) were investigated. The stability constant of NM·CDM-AM and MBC·CDM-AM complexes at 303 K were of 13,446.06 M^−1^ and 2595.3 M^−1^, respectively. The complexes were characterized using phase solubility diagrams, NMR spectra and FT-IR spectra. The analysis of the biological activities of these two complexes indicated that they possessed enhancing fungicidal activities compared to NM and MBC alone.

## 1. Introduction

β-cyclodextrin (β-CD) produced from starch by enzymatic conversion [1], and made up of seven glucose molecules bonded together forming a ring [2].can form inclusion complexes in its inner cavity with hydrophobic drugs, which can increase its solubility in water. However, the solubility of β-CD is very low, which is 1.6 mmol/L [3,4]. Low water-solubility of β-CD limits its application as embedding medium for hydrophobic drugs [5–7]. So that improving the water solubility of β-CD becomes one of the researching highlights in the β-CD modification. Preparing the water soluble β-CD polymer is one way to increase β-CD solubility.

In our former research reported that β-cyclodextrin-acrylamide (CDM-AM) copolymer was synthesized by β-CD maleate (CDM) and acrylamide using potassium persulfate (K_2_S_2_O_8_) as the initiator. The copolymer could increase the solubility of Methyl-2-benzimidazolecarbamate (MBC) and Natamycin (NM) and the stability constant of MBC·CDM-AM complex and NM·CDM-AM complex were 3000.89 M^−1^ and 10,725.45 M^−1^, respectively [8]. However, that method needs adding chemical initiator. In this research, γ-ray was used to initiate the copolymer reaction.

Radiation polymerization is one of the important research fields in radiation chemistry. Compared to the traditional technology of polymerization method, it has many advantages in improving the polymer performance, such as high grafting rate, no harmful chemical residue, no selectivity to substance and so on. Theoretically radiation polymerization technology can be applied to any monomer system [9]. Irradiation polymerization has been used in carbohydrates hydrogel preparation and its swelling and phenol absorbing properties were also studied [10]. However, irradiation polymerization has not been reported in the preparation of cyclodextrin polymer before.

The aim of this work was to synthesize the water soluble cyclodextrin copolymer by radical polymerization initialized by γ-ray, and form the inclusion complexes with hydrophobic drugs, NM and MBC. And the difference of CDM-AM copolymers synthesized by irradiation and chemical initiator were also compared in this article. The synthetic complexes were systematically characterized using size exclusion chromatography (SEC), phase solubility diagrams, NMR spectra, FT-IR spectra. Additionally, the water solubility and fungicidal activity of NM, MBC, NM·CDM-AM and MBC·CDM-AM were investigated.

## 2. Experimental

### 2.1 Materials

Maleic acid (MA) was purchased from Aladdin Industrial Corporation. NM and MBC were purchased from TCI. Sodium hypophosphite monohydrate (SHP), β-CD, 4-methoxyphenol, and acrylamide (AM) were purchased from China National Pharmaceutical Group Corporation. Dextran standard substance (relative molecular mass of 40,000) was purchased from Sigma-Aldrich (USA). All reagents were analytically pure unless otherwise noted.

### 2.2 Methods

#### 2.2.1 Preparation of the CDM-AM copolymer

(1) Synthesis of CDM

CDM was prepared via the semi-dry reaction method which was reported in our former research [11]. β-CD (6 mmol) was mixed with maleic acid (24 mmol), SHP (6 mmol) and 4-methoxyphenol (0.6 mmol) in a pressure bottle. SHP was the catalyst, and 4-methoxyphenol was polymerization inhibitor to protect the double bond. Then certain amount of water was added in the pressure bottle, and the M/L mass ratio was 1:0.6. The pressure bottle was placed in a circulating air oven at 110 ^◦^C for3.5 h. The reaction mixture was cooled down to room temperature and crystallized at 4 ^◦^C in a refrigerator, and the crystals were purified by washing with 95% ethyl alcohol, followed by drying at 60 ^◦^C for 24 h.

(2) CDM-AM copolymer synthesized by irradiation

CDM-AM was synthesized using γ-ray irradiation. The reaction mechanism for the synthesis of CDM-AM was shown as below:

Previous research indicates that when the hydrone is irradiated by γ-ray, molecules accept the energy of radiation and then are ionized and excited to generate the hydrone ions free radical (H_2_O^+^) and hydrone molecules free radical (H_2_O·). Meanwhile, the H_2_O^+^ reacts with other molecules to generated H_3_O^+^ and hydroxyl radical (OH·). There is also a part of the water molecules directly ionized and decomposed into hydrogen radical (H·) and hydroxyl radical (OH·) [12]. Therefore, the result of radiolysis reaction process of water molecular is the formation of three free radicals: H·, OH· and eaq^−^. OH· is a strong oxidant, which could induce the polymerization of vinyl monomer.

The genenrated hydroxyl radical (OH·) attack the vinyl group of CDM and AM to initiate vinyl polymerization.

**Figure:**
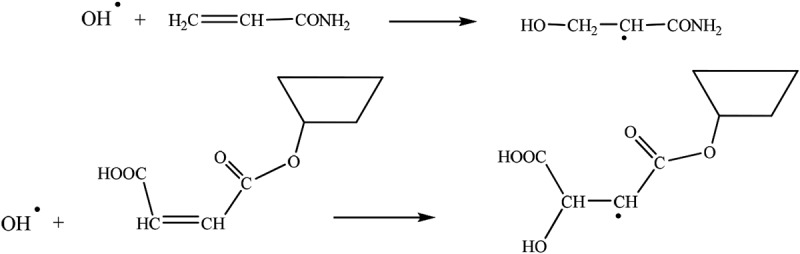

Subsequent addition of monomer molecules to the initiated chain ultimately leads to the formation of the CDM-AM copolymer.

**Figure:**
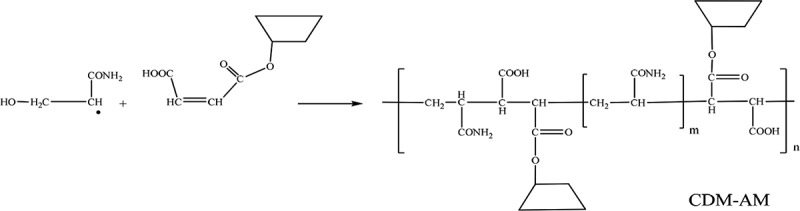

**Figure:**
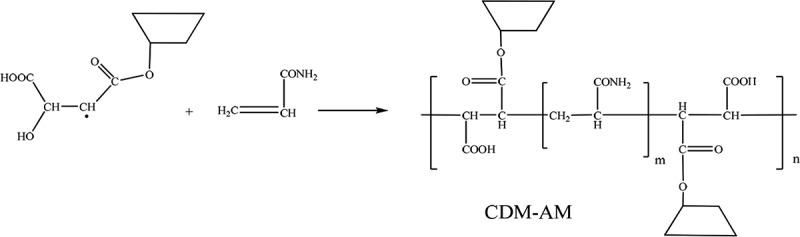

Polymerization of AM also occurs as a side reaction. Due to the steric hindrance of the β-CD ring in the CDM molecule, CDM cannot polymerize with itself.

**Figure:**
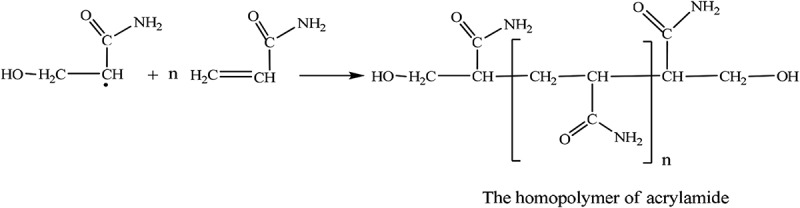

Thus, these experiments are designed to discover the major factors controlling the magnitude of polymerization. The synthetic steps leading to the formation of the CDM-AM copolymer are shown as follows:

a) A specific amount of CDM (3.6 g) and AM (3.6 g) was weighed and placed in 50 mL conical flasks with a stopper. 10 mL of double distilled water (deoxygenated by purging through nitrogen gas) was added to the flask. The bottle of reaction mixture was handled with ^60^Co γ-ray radiation to start the radiation copolymerization (Irradiation was conducted at the institute of agricultural products processing irradiation center, Chinese academy of agricultural sciences), the temperature was 25 ^◦^C, dose range was from 2 to 10 kGy, and the dose rate was 0.5 kGy/h.
b) After reaction completion, the reaction mixture was transferred to a beaker. The reaction flask was washed three times with 5 mL of double distilled water, and the polymer was precipitated by slowly adding 80 mL of ethanol to the beaker. The solution was then stirred until it turned clear. The supernatant was discarded and the sticky solid particles were collected and washed three times using 30 mL of anhydrous ethanol. The filter cake was collected and dried at 60°C in an oven for 6 h and weighed. Polyacrylamide (without CDM) was prepared using the same method described above.

#### 2.2.2 The product yield and molecular weight of the CDM-AM copolymer

The yield of product (YP) was calculated as follows:

(1) $$YP(\%)=\frac{W_{0}}{W_{1}+W_{2}}\times 100\%$$

where W_0_ is the weight of the polymer and W_1_ + W_2_ represents the added amounts of CDM and AM, respectively.

The molecular weight of the CDM-AM copolymer was determined by combining a multi-angle laser light scattering instrument with size exclusion chromatography (MALLS-SEC, L-2130, HITACHI, Japan) and the method of molar mass calculation was based on MALLS with dn/dc, using dextran as the standard. The CDM-AM copolymer (1 mg/mL) was dissolved by moving phase (0.1 mol/L NaNO_3_ aqueous solution containing 0.2‰ NaN_3_) and filtered using a 0.45 μm membrane before SEC injection. The injection volumes were 200 μL, the UV detector wavelength was 280 nm, the differential refractometer detector wavelength was 690 nm and the flow rate was 0.50 mL/min.

#### 2.2.3 Preparation of the inclusion complex with CDM-AM copolymer

MBC (0.0478 g, 0.25 mmol) and NM (0.1664 g, 0.25 mmol) were mixed with the CDM-AM copolymer (1.013 g, M_w_ = 43,300), respectively, before adding 20 mL of double distilled water. The reaction mixture was then shaken at a speed of 120 r/min for 3 days. The turbid liquid was filtered through a 0.45 μm hydrophilic membrane filter using a syringe. Lastly, the filtrate was dried in a vacuum freeze drier.

#### 2.2.4 Phase solubility studies

The phase solubility studies were performed according to a method reported by Higuchi and Connors [13]. NM (50 mg) and MBC (10 mg) were added in excess to aqueous solutions (10 mL) containing different concentrations of CDM-AM copolymer (0.23, 0.46, 0.69, 0.92, and 1.15 mmol/L). The weight-average molecular weight (M_w_) of CDM-AM copolymer was 43, 300. The stoppered conical flasks were sealed with a plastic film to avoid water evaporation and then shaken at 303 K, 313 K, and 323 K for 3 days. After equilibrium was established, the suspensions were filtered through a 0.45 μm hydrophilic membrane filter by using a syringe. The resulting filtrates were diluted and the concentrations of NM and MBC were analyzed at 303 and 281 nm using a UV-Vis spectrophotometer (UV-1800, SHIMADZU, Japan). The linear regression equations used to determine the concentrations of NM and MBC were as follows:

A_NM_ = 0.0546 C (mg/mL) + 0.0031 (R^2^ = 0.9992)

A_MBC_ = 0.0967 C (mg/mL) + 0.0003 (R^2^ = 0.9999)

The apparent stability constant Kc was calculated from the linear line obtained from the phase solubility diagram.

(2) $$K_{c}=\frac{Slope}{S_{0}(1-Slope)}$$

where S_0_ is the intrinsic solubility of NM and MBC in redistilled water in the absence of the CDM-AM copolymer.

#### 2.2.5 UV spectroscopy

The aqueous solution concentration of CDM-AM, acrylamide and β-cyclodextrin maleate (CDM) were 1 mg/ml, 2 μg/ml and 5 μg/ml, respectively. The UV spectroscopy was detected by using ultraviolet-visible spectrophotometer (UV – 1800, Shimadzu, Japan), interval wavelength of scanning was 0.1 nm, and scanning speed was fast mode.

#### 2.2.6 NMR spectroscopy

^1^H NMR spectra of CDM, NM, MBC, CDM-AM copolymer and the complexes of NM·CDM-AM and MBC·CDM-AM were collected at 25°C by a Bruker-500 spectrometer (AVANCE III, Bruker, Switzerland). All NMR samples were prepared in D_2_O, with the exception of NM and MBC, which were prepared in deuterated methanol.

#### 2.2.7 Ft-ir

FT-IR spectra were collected using a FT-IR Spectrometer (Tensor-37, BRUKER, Germany). Specifically, the sample was ground with KBr (about 200–400 mg) into a fine powder, placed into the sampling cup, smoothed, and compressed into a transparent flake by using a tablet machine. At this point, the sample was placed in the beam path and the FT-IR spectrum was obtained.

#### 2.2.8 Thermal analysis

Thermo-gravimetric analysis was performed using a TG/DTA thermal analyzer (Pyris-115, Perkin Elmer, USA) with the following experimental conditions: nitrogen atmosphere (25 mL/min), 10°C/min heating rate, and a scanning temperature range from 40°C to 450°C.

#### 2.2.9 Scanning electron microscope

A scanning electron microscope (SEM) examination was carried out by counting the CDM-AM copolymer samples on sub with double stick adhesive tape and coated with gold in a S150A sputter coater unit (Edwards, UK), the gold film thicknees was 150°A, then viewed in a JEOL JSM-6700F electron probe micro-analysis.

#### 2.2.10 Bioassay of fungicidal activity

The fungicidal activities of the NB, MBC, NM·CDM-AM and MBC·CDM-AM against A. niger (A. niger 04523 was purchased from Institute of Microbiology, Chinese Academy of Sciences) were determined using the Czapek Dox Agar method [8].

Equilibrium turbid liquid prepared at 303 K in section 2.2.2 were filtered through a 0.45 μm hydrophilic membrane filter. About 15 μL of each of the filtrate, the inclusion complexes of NM·CDM-AM and MBC·CDM-AM were added to paper disks, respectively. Within 5 min, the drug-loaded paper disks (6 mm in diameter) were placed on an inoculated plate and incubated overnight at 30°C. The fungicidal activity was determined by the size of bacterial clearance, which was measured by calipers across an average diameter.

#### 2.2.11 The determination of residual acrylamide monomer in CDM-AM copolymer

Acrylamide standard substance solution (1 mg/ml) was diluted by gradient, and the final concentration of acrylamide was 0.25, 0.50, 1.00, 5.00, and 10.00 μg/mL, respectively. Then they were analyzed by selected HPLC conditions. The standard curve of acrylamide was drawn with concentration as the abscissa and peak area as the ordinate. Adequate amount of the CDM – AM polymers were weighed accurately, dissolved by 10% (volume fraction) acetonitrile water solution, and configured to concentration of 1 mg/ml. The sample was filtered by 0.45 μm microporous membrane, analyzed by selected HPLC conditions. Chromatographic conditions were as follows: the stationary phase was Innoval – C18 column (50 mm × 4.6 mm, 5 μm) from agela technologies. The mobile phase was a mixture of acetonitrile (A, 15%) and water (B, 85%). The flow rate was 1.0 mL/min and the injection volume was 10.00 μL. The UV detector wavelength was 200 nm and column temperature was 25°C.

## 3. Results and discussion

### 3.1 Synthesis of the CDM-AM copolymer

**Figure 1.**
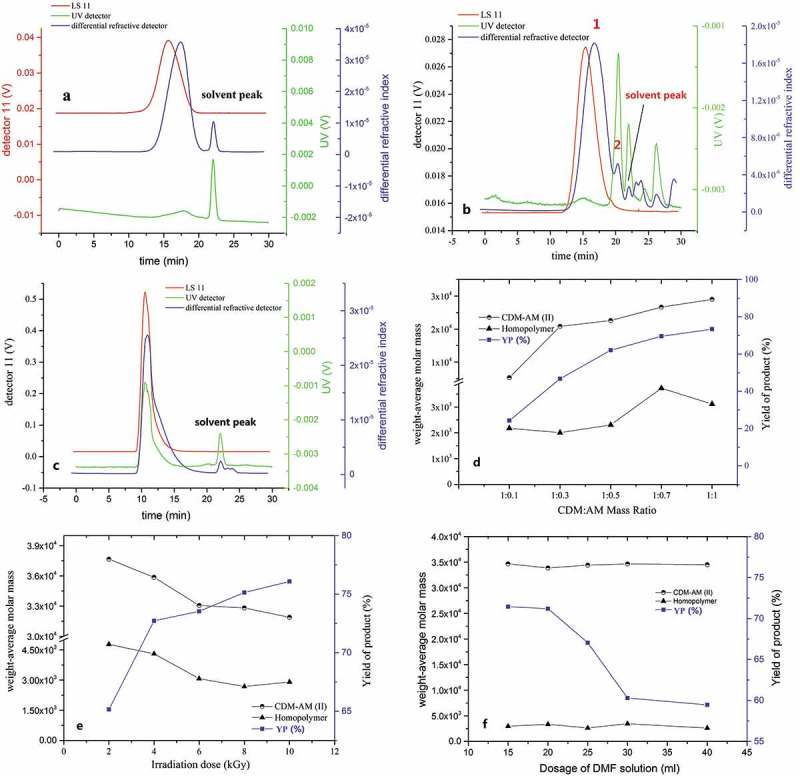
Gel Permeation Chromatography (GPC) of the Dextran (a), CDM-AM copolymer (b), AM homopolymer (c) and the factors affecting the preparation of CDM-AM copolymer (d-f).

The Gel Permeation Chromatography (GPC) of the CDM-AM copolymer is shown in Figure 1b. Two peaks were observed before the solvent peak, and only the second peak (peak 2) demonstrated ultraviolet absorbance. The AM homopolymer is known to have greater amounts of amido bonds. Literature shows that amido bonds have an ultraviolet absorption peak at 280 nm. The GPC of the AM homopolymer also emphasized this point (shown in Figure 1c). Based on this, it was determined that peak 2 was the homopolymer of AM. In general, carbohydrates do not absorb or only weakly absorb ultraviolet light at 280 nm, similar to the dextran standard (Figure1a). Thus, peak 1 was assigned as the CDM-AM copolymer. The GPC of the synthetic CDM-AM copolymer (Figure 1b) also proved that the CDM-AM copolymer was successfully synthesized, with the polymerization of AM occurring as a side reaction during the chemical synthesis.

In this study, the weight-average molar mass of CDM-AM copolymer increased as AM was consumed in the reaction, and the value for the yield of the CDM-AM copolymer became stable when the CDM:AM mass ratio was 1:1. (Figure 1d). The irradiation dose had significant influence on the weight-average molar mass and yield of the CDM-AM copolymer and homopolymer. The yield of CDM-AM increased with irradiation dose, however, weight-average molar mass decreased with irradiation dose (Figure 1e). This result was due to the faster generation of free radicals at higher irradiation, leading to an increase in the rate of the termination reaction. The dosage of DMF solution had no significant influence on the weight-average molar mass the weight-average molar mass of the CDM-AM copolymer but the yield of product was decreased as the increasing of dosage of DMF solution (Figure 1f). This result was due to the self-polymerization of acrylamide monomer.

These results indicate that the optimized preparation conditions for the CDM-AM copolymer are as follows: CDM:AM mass ratio of 1:1; irradiation dose of 4 kGy; and using 20 mL of DMF water solution. The yield rate of CDM-AM was 75% in grams using these synthetic conditions.

### 3.2 Phase solubility studies

**Figure 2.**
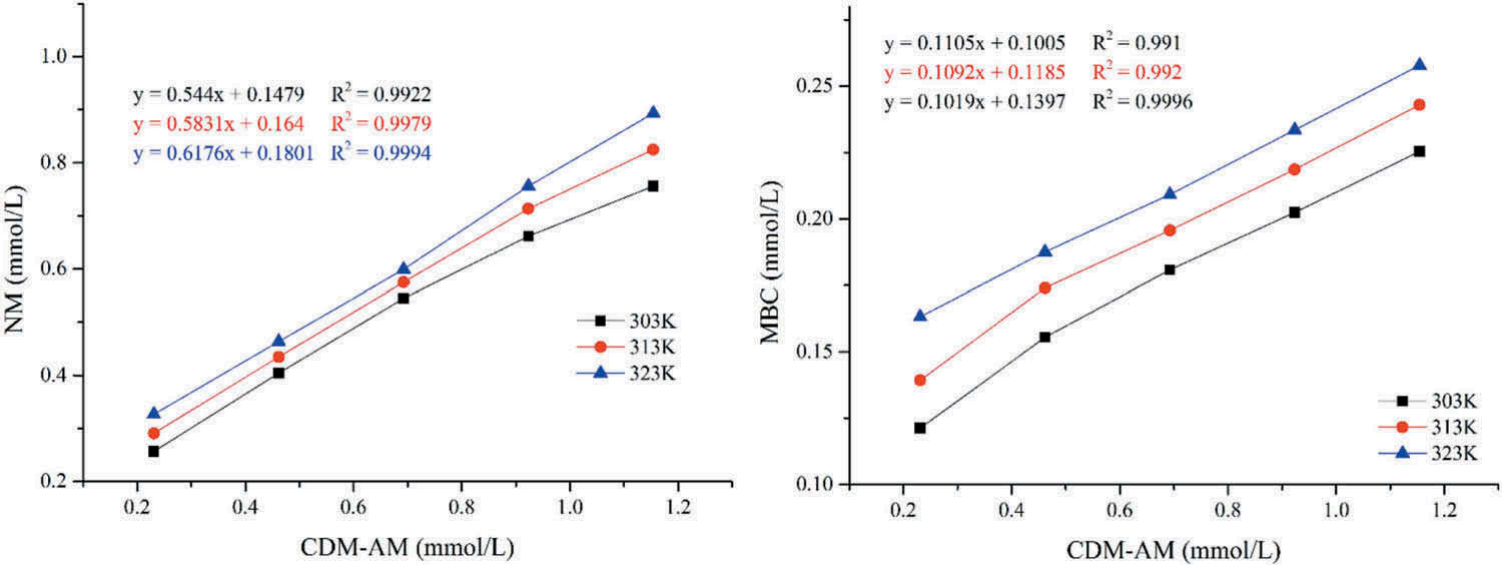
Phase solubility diagram of NM (a) and MBC (b) in the presence of CDM-AM copolymers.

Phase solubility diagrams have been used extensively to investigate the solubility of particular drugs and agrochemicals in the presence of CDs [14]. The phase solubility diagrams of NM and MBC in the presence of CDM-AM copolymers are presented in Figure 2.

The solubility of NM and MBC increased linearly with increasing concentrations of CDM-AM. Thus, the phase solubility diagrams of NM·CDM-AM and MBC·CDM- AM could be classified as type A_L_ [13], which was same with the complex of CDM-AM synthesized by chemical method. The values of the apparent stability constant Kc, calculated according to Eq. 2, were shown in Table 1. Compared with the water solubility of free NM and MBC, the solubility of NM and MBC increased 8.5-fold and 4.7-fold, respectively, in the presence of 1.15 mmol/L CDM-AM at temperature of 303 K. The concentration of NM and MBC in CMD-AM copolymer water solution also increased with temperature (Table 1). But their solubility in CDM-AM synthesized by irradiation were lower than that synthesized by chemical initiator. This was because γ-ray could cause the degradation of cyclodextrin part in CMD-AM copolymer. According to the apparent stability constant shown in Table 1, the NM·CDM-AM complex was more stable than the MBC·CDM-AM complex in an aqueous solution. However, there was a decrease in the Kc values of the NM·CDM-AM and MBC·CDM-AM complexes as the temperature was increased, and the S_0_ values of NM and MBC were increased as the temperature increasing. In addition, the Kc of NM was higher than that of MBC at any temperature (Table 1), indicating that the NM·CDM-AM complex was more stable than MBC·CDM-AM complex. Because of the increased molecular size of NM compared to MBC, NM was more suitable for the β-CD ring of the CDM-AM copolymer. These results suggested that the CDM-AM copolymer was a good biosorbent for NM and MBC from aqueous solutions and could be used as a drug carrier for NM and MBC.

**Table 1.** The apparent stability constant K_c_ and S_0_ of the NM·CDM-AM complex and the MBC·CDM-AM complex at different temperature.

Temperature	303 K	313 K	323 K
K_c_ of NM (M^−1^)	13,446.06	8672.00	7840.49
S_0_ of NM (mmol/L)	0.0887	0.144	0.206
K_c_ of MBC(M^−1^)	2595.30	2028.19	1429.51
S_0_ of MBC (mmol/L)	0.0479	0.0604	0.0794

### 3.3 Characterization of CDM-AM copolymer and complexes with NM and MBC

(1) UV spectra

**Figure 3.**
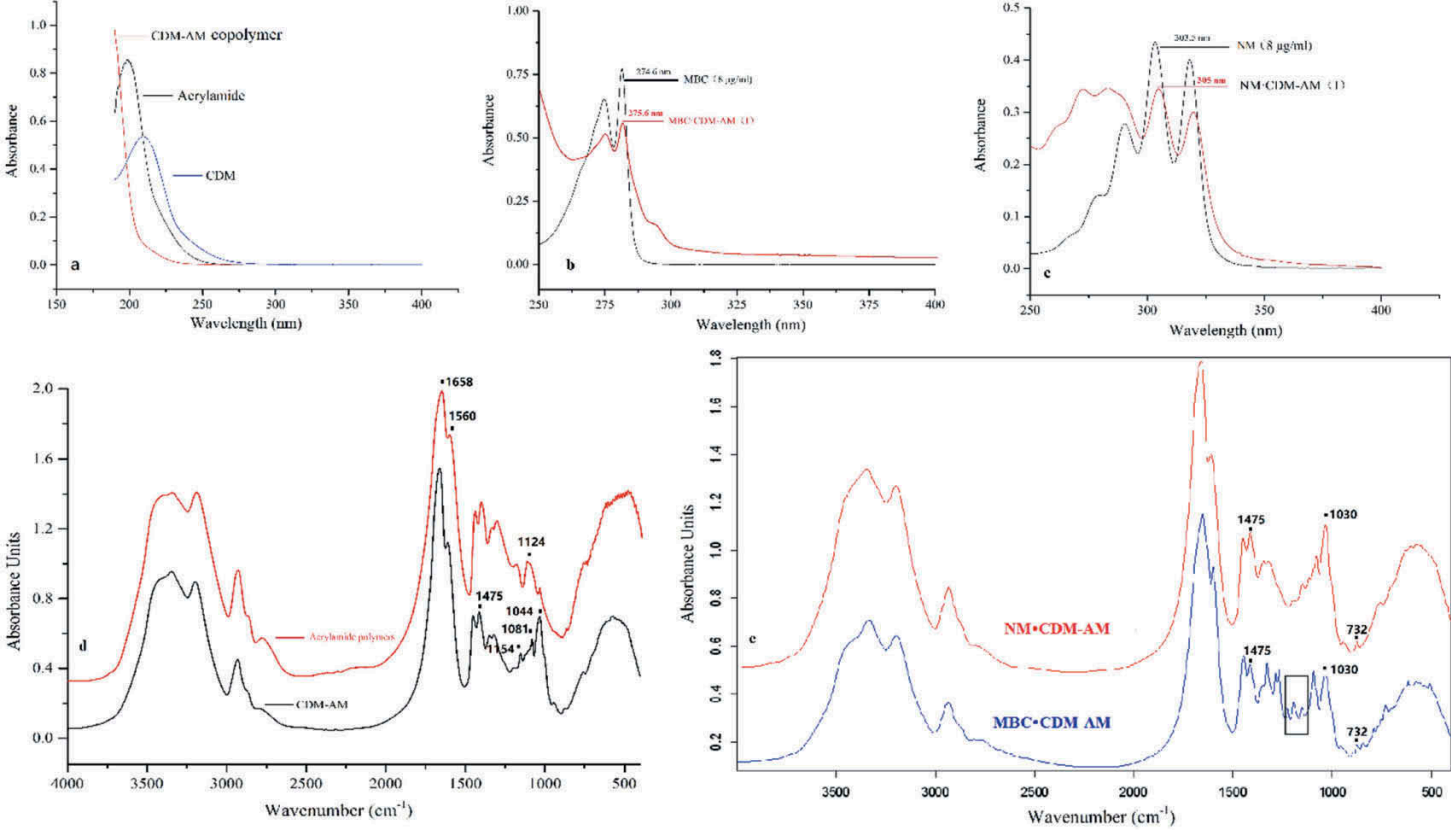
UV spectra of CDM, CDM-AM, Acrylamide (a), MBC, MBC·CDM-AM (b), and NM, NM·CDM-AM (c); FT-IR spectra of polyacrylamide, CDM-AM copolymer (d); FT-IR spectra of inclusion complex MBC·CDM-AM, and inclusion complex of NM·CDM-AM (e).

CDM and acrylamide had absorption peaks under wavelength 210 nm and 205 nm, respectively. There was no ultraviolet absorption peak in the UV spectra of CDM-AM polymer at the wavelengths mentioned above (Figure3a), which indicated that there was intermolecular polymerization between CDM and acrylamide due to the double bond in the CDM and acrylamide being damaged. UV spectra was confirmed that the CDM-AM polymer was synthesized. Ultraviolet spectrum of carbendazim and MBC·CDM-AM was shown in Figure3b. MBC had biggest ultraviolet absorption at 274.6 nm, but maximum absorption of MBC·CDM-AM complex redshifted to 275.6 nm. Similar phenomenon was observed in the UV spectrum of NM and NM·CDM-AM complex. The biggest ultraviolet absorption of NM was at 303.5 nm, but the biggest ultraviolet absorption of NM·CDM-AM complex redshifted to 305 nm (Figure3c). The UV spectrums indicated that CDM-AM could form complexes with MBC and NM.

(2) FT-IR spectra

The FT-IR spectra of polyacrylamide and the CDM-AM copolymer were presented in Figure 3d. For the CDM-AM copolymer, absorption bands were observed around 3201 (ν NH_2_), 1658 (ν C = O), and 1560 cm^−1^ (δ NH_2_), as well as 1154, 1081, and 1044 cm^−1^ (ν C-OH of cyclodextrin) (Figure 3d). The absorption peak at 1642 cm^−1^ for the stretching vibration of the alkene (C = C) bond in CDM also disappeared [8]. These results indicate that the CDM-AM copolymer was synthesized. For polyacrylamide, a peak at 1124 cm^−1^ (corresponding to the C-N-C stretch, Figure 3d) was observed, suggesting that there is intermolecular cross-linking in the polyacrylamide molecule. The absence of any signal at 1124 cm^−1^ (corresponding to the C-N-C stretch) indicates that the CDM-AM molecule could be a linear high-molecular polymer. The absorption peaks at 2865 and 2780 cm^−1^ also disappeared in the spectrum for the CDM-AM copolymer compared to the polyacrylamide spectrum, further confirming the successful synthesis of the CDM-AM copolymer.

The variation in the shape, shift, and intensity of the FT-IR absorption peaks for the guest or host can provide enough evidence for inclusion [15]. The FT-IR spectra of the inclusion complex MBC·CDM-AM and the inclusion complex NM·CDM-AM were presented in Figure3e. In the FT-IR spectrum of the MBC·CDM-AM complex, there were characteristic absorption peak of MBC during 1250 cm^−1^ and 1100 cm^−1^. However, the band corresponding to the C = O stretching vibration of the ester at 1712 cm^−1^ disappears in the complex. The band at 1044 cm^−1^, corresponding to the C-OH stretching vibration of the cyclodextrin, shifted to 1030 cm^−1^. Meanwhile, the intensities of the C-H bending vibration of the benzene ring and conjugated double bond at 732 cm^−1^ increased, and the NH of the secondary amide at 1475 cm^−1^ was decreased [8]. Therefore, the FT-IR spectra confirm the formation of the inclusion complex, specifically with the benzene ring of MBC included into the CDM-AM cavity. Moreover, In the FT-IR spectrum of the NM·CDM-AM complex (Figure 3e), the band corresponding to the C = O stretching vibration of the ester in NM at 1715 cm^−1^, also disappeared in the complex [8]. The band corresponding to the C-OH stretching vibration of cyclodextrin at 1044 cm^−1^ shifted to 1030 cm^−1^. Therefore, FT-IR spectra confirm that the inclusion complex NM·CDM-AM was formed and that NM was included into the CDM-AM cavity.

(3) NMR spectra

**Figure 4.**
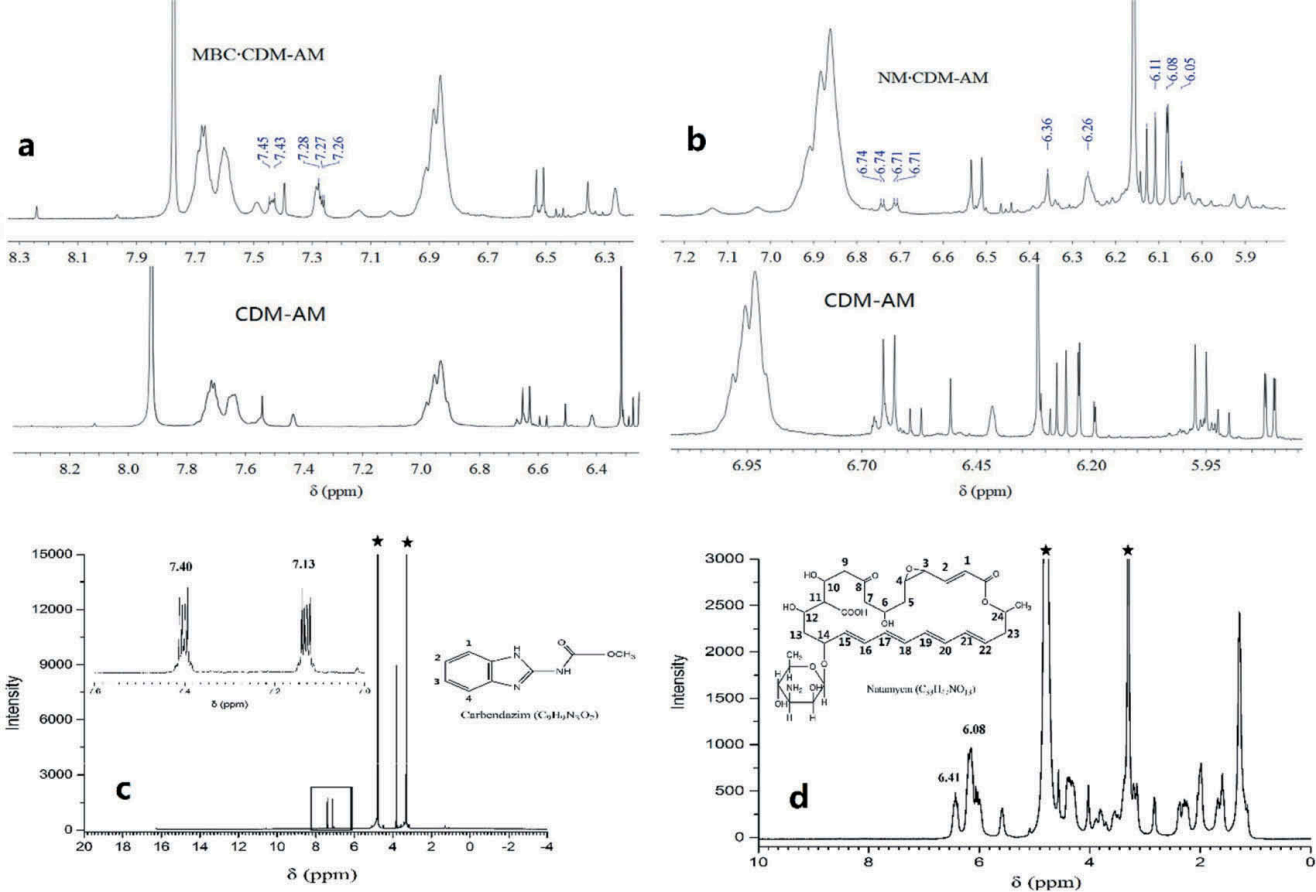
^1^H NMR spectrum of the CDM-AM copolymer (a); ^1^H NMR spectra of the CDM-AM copolymer and its complexes with NM and MBC (b); ^1^H NMR spectrum and molecular structure of MBC (c); ^1^H NMR spectrum and molecular structure of NM(d).

When comparing the ^1^H NMR spectra for NM, the CDM-AM copolymer and their complex, the 1H chemical shift of the conjugated double bond in NM was divided from one multiplet peak to several singlet peaks between 6.0 ppm and 6.5 ppm (Figure 4d). A doublet corresponding to the carboxylic acid group at 6.74 ppm (11-H) was also observed. Peaks corresponding to the vinyl proton of the unsaturated ester at 6.48 ppm and 6.39 pm (Figure4b) also appear in the 1H spectrum for the NM·CDM-AM complex (Figure 4b). These results suggest that the chemical shifts of the two H atoms in the vinyl group become divided when the NM·CDM-AM complex is formed. In summary, we determined that the NM·CDM-AM complex was successfully formed.

Similar to NM, we found that the 1H chemical shifts of the benzene rings in MBC also shifted from high field to low field in the MBC·CDM-AM complex. The ^1^H chemical shifts of the benzene rings in MBC were observed at 7.13 ppm (2-H and 3-H) and 7.40 ppm (1-H and 4-H) (Figure 4c), while the 1H chemical shifts for the benzene rings of the MBC·CDM-AM complex were observed at 7.44 ppm (1-H and 4-H) and 7.27 ppm (2-H and 3-H) (Figure 4a). The peak pattern of 2-H and 3-H in the benzene ring of MBC also changed, indicating that the active site of MBC within the CD ring was located at the 2-H and 3-H atoms in the benzene ring of MBC. These results also suggest that the MBC·CDM-AM complex was successfully formed. In conclusion, MBC and NM were both able to form inclusion complexes with the CDM-AM copolymer.

### 3.4 Thermo-gravimetric analysis (TGA) and SEM analysis

**Figure 5.**
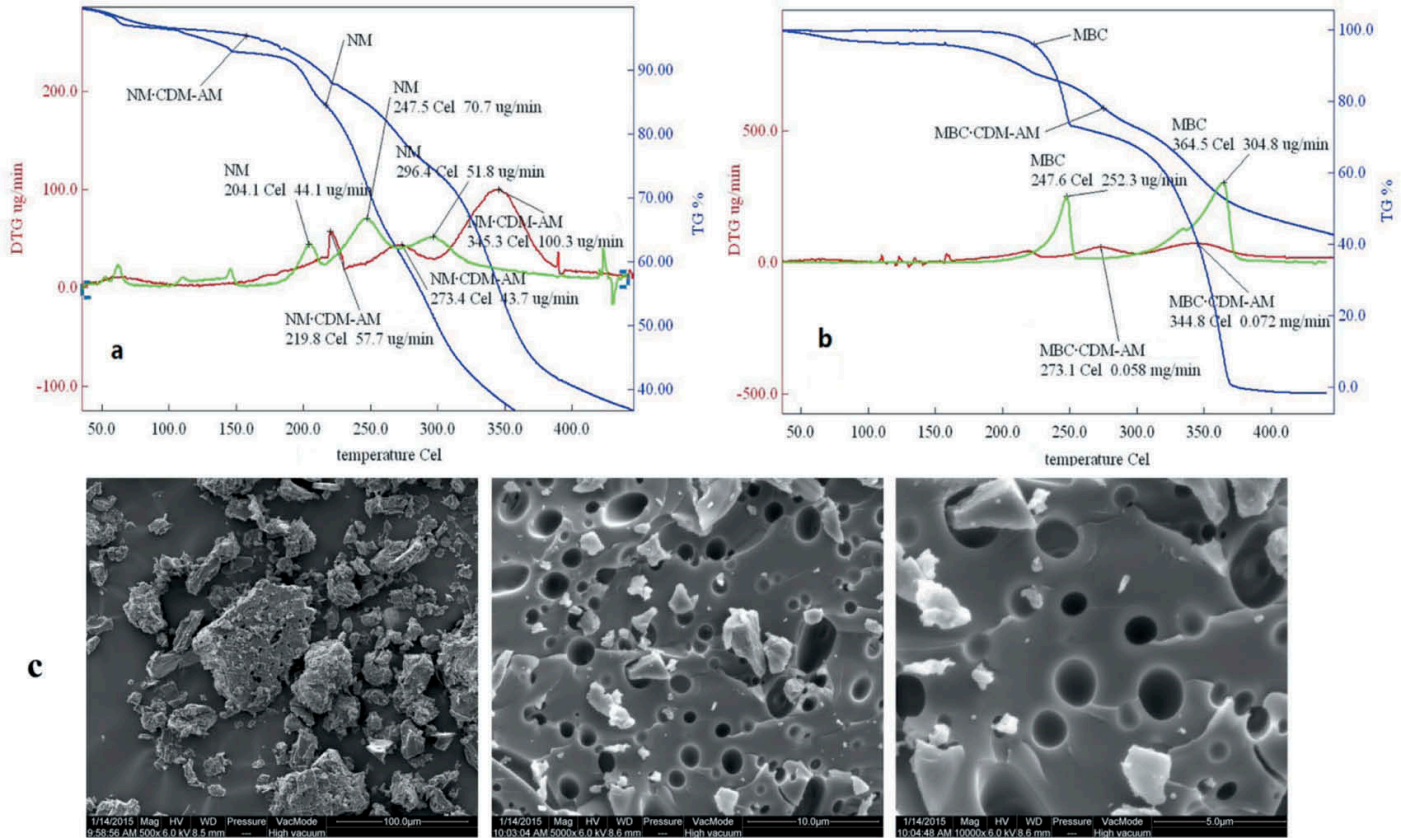
TGA curves of CDM and the CDM-AM copolymer (a: TG and DTG of the CDM-AM copolymer and its complex with NM; b: TG and DTG of the CDM-AM copolymer and its complex with MBC) and SEM picture of CDM-AM copolymer (c).

Thermo-gravimetric analysis (TGA) was carried out to determine the thermal properties and stability of the new biomaterials. Figure 5 shows the thermo-gravimetric (TG) curves and the first derivative TG (DTG) traces of CDM-AM copolymer and its complex with NM (Figure5a) and MBC (Figure5b). The TG curves show that the thermal stability of the NM·CDM-AM complex was Higher than that of NM. The initial degradation temperature of the NM embedding in CDM-AM copolymer was 219.8 ºC, whereas NM was 204.1 ºC (Figure 5a). The initial degradation temperature of the MBC embedding in CDM-AM copolymer was 273.1 ºC, whereas MBC was 247.6 ºC (Figure 5a). Based on the DTG curves of the drugs complex with CDM-AM copolymer, the maximum weight losses for the NM·CDM-AM complex and MBC·CDM-AM complex were 57.7 μg/min at 175 ºC and 58 μg/min at 273.1 ºC, respectively. The TGA results also provide further proof that NM and MBC can be formed the complex with the CDM-AM copolymer and the cyclodextrin ring of CDM-AM copolymer could protect the drugs embedding in it.

According to scanning electron microscopy (SEM) picture of CDM-AM polymer, the structure of CDM-AM copolymer was porous structure (as shown in Figure5c), which was obvious different in the crystalline structure of CDM and acrylamide, and this can be used as the synthesis evidence of CDM-AM polymer one of. At the same time, the cellular structure of polymer availed the water molecules to enter the inside of polymer, which could increase the dissolution rate of polymer in the water. That’s why the CDM – AM copolymer had a good solubility in water.

### 3.5 Fungicidal activity

**Figure 6.**
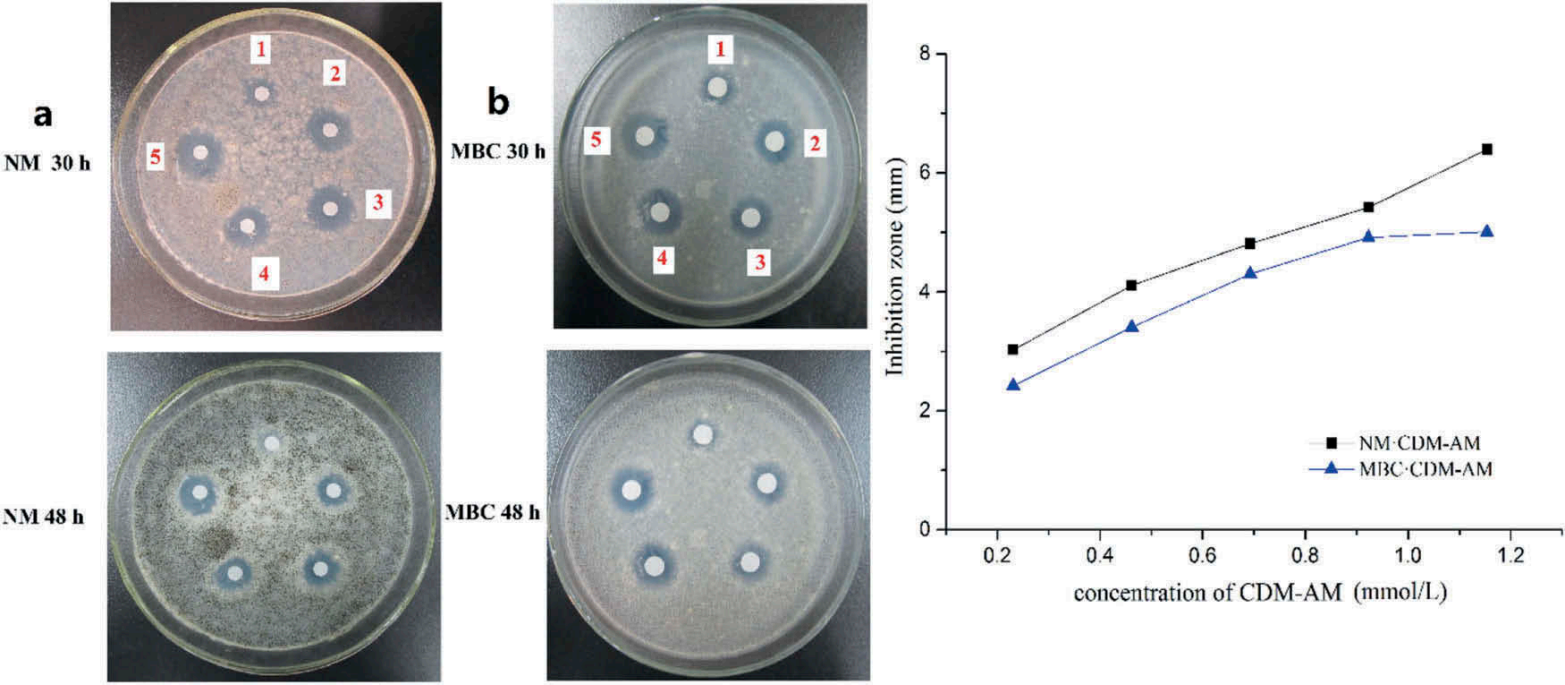
The fungicidal activity for A. niger of the inclusion complex NM·CDM-AM (a1–5) and the inclusion complex of MBC·CDM-AM (b1–5).

NM and MBC were chosen as research object based on their wide application and effectiveness in the field of agriculture and food storage. The zones of inhibition were defined as the maximum distances between the test disk and the fungal growth edge. The zones of inhibition for NM·CDM-AM (a1-a5) and the zones of inhibition for MBC·CDM-AM (b1-b5) are shown in Figure 6. According the inhibition zones of the complexes, the growth of hypha was significantly inhibited, because the CDM-AM copolymer significantly improved the water solubility and the bioavailability of NM and MBC. The inhibition ability of the drugs also increased with concentrations of the CDM-AM copolymer (Figure 6c). Compared to free NM and MBC, the NM and MBC complexes demonstrated a 1.92- and 1.73-fold increase in fungicidal activity at a concentration of 1.15 mmol/L (CDM-AM copolymer), respectively. The zone of inhibition gradually decreased over time, therefor the complexes had slow-releasing potential. Although the concentration of NM in the CDM-AM solution was higher than that of MBC, the inhibition zones showed no significant differences from each other, probably because the NM·CDM-AM copolymer complex was more stable than that of MBC·CDM-AM. Together, these results may provide useful information for the facile application of both NM and MBC.

Compared to the fungicidal ability of the complex of CDM-AM copolymer synthesized by chemical method with NM and MBC [8], the fungicidal ability of the complex NM·CDM-AM and MBC·CDM-AM were all decreased in this paper. That’s because the radiation could degrade the cyclodextrin ring of the CDM-AM copolymer and there was less absorption part in the copolymer synthesized by irradiation.

### 3.6 Residual amount of acrylamide

**Figure 7.**
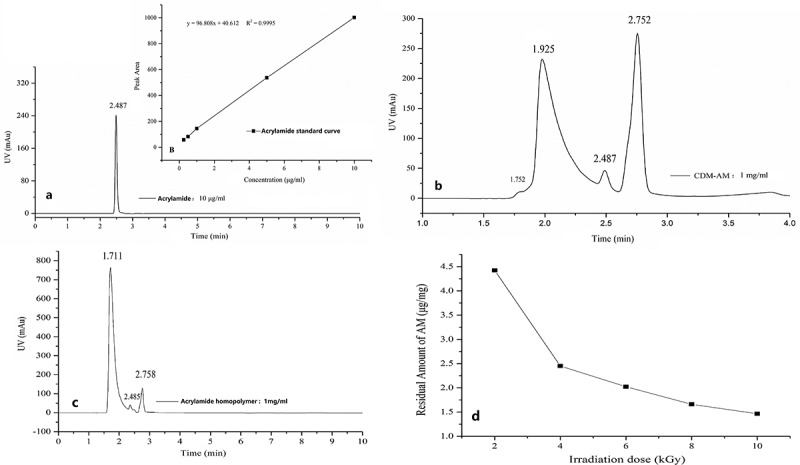
The HPLC spectra and standard curve of acrylamide (a); HPLC spectra of CDM-AM (b) and Acrylamide homopolymer (c); Residual amount of acrylamide in CDM-AM copolymer (d).

Based on HPLC spectra of acrylamide homopolymer, acrylamide can form two kinds of homopolymer under the γ-ray irradiation, of which retention time was 1.711 min and 2.758 min (Figure7c). Hence in the HPLC spectra of CDM-AM copolymer, absorption peaks at retention time of 2.752 min was PAM homopolymers, and the absorption peak at retention time of 1.925 min was CDM-AM polymer (as shown in Figure7b). HPLC spectra of CDM-AM polymer also further confirmed the synthesis of CDM-AM polymer. Residual amount of acrylamide under different irradiation dose was calculated by the standard equation shew in Figure7a. With the increase of irradiation dose, the residues amount of acrylamide gradually reduced (Figure7d).

## 4. Conclusion

In this work, the CDM-AM copolymer was prepared from AM and CDM using γ-ray as an initiator. The preparation conditions for the CDM-AM copolymer were as follows: CDM:AM mass ratio of 1:1; irradiation dose of 4 kGy; and using 20 mL of DMF water solution. The yield rate of CDM-AM was 75% in grams using these synthetic conditions. The complexes of NM·CDM-AM and MBC·CDM-AM were also prepared, with apparent stability constants at 303 K of 13,446.06 M^−1^ and 2595.30 M^−1^, respectively. The NM·CDM-AM and MBC·CDM-AM complexes demonstrated significantly improved water solubility and NM/MBC bioavailability, providing a promising approach for the more straightforward application of NM and MBC. As the increase of irradiation dose, the residues amount of acrylamide gradually reduced, however, the high irradiation dose also can cause the degradation of β-cyclodextrin which could reduce the CDM-AM polymer’s solubilization effect on hydrophobic drugs.
